# Comparative analysis of monoterpene indole alkaloid composition and genotypic variation in Thai *Mitragyna speciosa*

**DOI:** 10.3389/fpls.2026.1821609

**Published:** 2026-05-04

**Authors:** Katherine E. Ransden, Pavithra Ramachandria, Larissa C. Laforest, Siva Rama Raju Kanumuri, Abhisheak Sharma, Christopher R. McCurdy, Satya Swathi Nadakuduti

**Affiliations:** 1 Plant Molecular and Cellular Biology Program, Institute of Food and Agricultural Sciences, University of Florida, Gainesville, FL, United States; 2 The Horticultural Sciences Department, Institute of Food and Agricultural Sciences, University of Florida, Gainesville, FL, United States; 3 Department of Pharmaceutics, College of Pharmacy, University of Florida, Gainesville, FL, United States; 4 Department of Medicinal Chemistry, College of Pharmacy, University of Florida, Gainesville, FL, United States; 5 Genetics Institute, University of Florida, Gainesville, FL, United States

**Keywords:** DNA barcoding, Kratom, *Mitragyna speciosa*, monoterpene indole alkaloids, targeted metabolomics

## Abstract

*Mitragyna speciosa* (commonly known as kratom) is a tropical tree native to Southeast Asia, ethnobotanically used for pain relief and fatigue management. Kratom has gained popularity in Western countries for its dose-dependent stimulant and opioid-like effects, and its reported use in the self-treatment of opioid withdrawal. Kratom leaves accumulate over 50 monoterpene indole alkaloids (MIAs) and oxindole alkaloids that have pharmaceutical significance, to which many of these effects are attributed. In this study, we characterized eight kratom accessions originating from Central and Southern Thailand which exhibited phenotypic variation in leaf morphology, including differences in shape, margins, and vein coloration. To authenticate and evaluate genotypic variation among these accessions, we employed DNA barcoding using four loci: the *nuclear ITS*, and three plastid barcodes including *rbcL, MatK*, and *trnH-psbA.* No polymorphisms were detected using *ITS* and *rbcL* barcodes. However, sequence analyses revealed insertion-deletion polymorphisms in *trnH-psbA*, and single nucleotide polymorphisms in both *MatK* and *trnH-psbA*, resolving intraspecific variation and separating the accessions into two distinct haplotypes. Targeted metabolite profiling was conducted using UPLC-MS to quantify 17 MIAs and oxindole alkaloids from both young and mature leaves across all kratom accessions. Mitragynine was the most predominant alkaloid in mature leaves, reaching up to 1.19% w/w of leaf dry mass, whereas juvenile leaves accumulated speciociliatine as the major alkaloid, at levels up to 1.14% w/w. Notably, strictosidine, the central precursor of MIA biosynthesis, was detected exclusively in juvenile leaves, which also exhibited significantly higher levels of upstream intermediates including corynantheidine, and iso-corynantheidine compared with mature leaves. In addition, juvenile leaves were dominated by 3*R* MIAs, whereas mature leaves accumulated higher levels of 3*S* MIAs. However, the relative distribution of 3*S* and 3*R* stereoisomers remained consistent across accessions. Under the conditions examined, leaf developmental stage exerted a greater influence on alkaloid composition than accession or haplotype variation. Despite visible distinction in leaf vein coloration, alkaloid profiles at maturity remained largely consistent across all accessions. The developmental chemotype patterns presented in this study provide a valuable framework for targeted breeding, metabolic engineering, and controlled cultivation strategies aimed at optimizing specific MIA profiles, particularly those of pharmacological interest.

## 1 Introduction

*Mitragyna speciosa* belongs to the Rubiaceae, also known as the coffee family, and is commonly sold as kratom. Traditionally, kratom leaves have been utilized by either chewing them directly or brewing them into tea. This usage is primarily for pain relief, combating fatigue, and easing symptoms of opioid withdrawal as mitragynine and its stereoisomer speciociliatine, present in *M. speciosa*, are partial human *µ*-opioid receptor agonists (Smith et al., 2023). *M. speciosa* trees reach heights of 25 meters, with trunk widths up to 0.9 m. The trunk generally grows straight and upright with smooth, gray bark. The leaves are glossy and dark green, with 12–17 pairs of veins, and an opposite phyllotactic arrangement (Ghazalli et al., 2021). Stipules are present at the base of each leaf pair. Leaf morphology varies, exhibiting ruffled, spiked, or smooth margins. The abaxial surface bears capitate glandular trichomes, whereas the adaxial surface is characterized by simple, unicellular trichomes. The vein coloration is variable, ranging from white to green to bright red, with pigmentation more pronounced on the abaxial surface. This vein coloration is commonly used to distinguish commercial *M. speciosa* products (Huisman et al., 2023). *M. speciosa* is native to central and southern Thailand, northern Malaysia, Sumatra, Borneo, the Philippines, and New Guinea (Ridsdale, 1978) encompassing considerable climatic and geographic variation.

Kratom leaves accumulate over 50 monoterpene indole alkaloids (MIAs) and oxindole alkaloids, many of which have pharmaceutical significance. Mitragynine, the predominant alkaloid in the mature leaves, acts as a partial agonist of the human *µ*-opioid receptor (Smith et al., 2023). All MIAs derive from the central precursor strictosidine, which is formed through a stereoselective Pictet–Spengler condensation between tryptamine and secologanin, catalyzed by strictosidine synthase. The formation of strictosidine marks the first committed step in MIA biosynthesis (Miettinen et al., 2014). The predominant MIAs in *M. speciosa* differ in stereochemistry at the C3 and C20 stereogenic centers, which contributes to variation in the experiences and physiological effects by individuals consuming kratom products with differing alkaloid compositions. MIAs are typically present in low concentrations within plants and display extensive structural and functional diversity, traits that likely evolved as defense mechanisms against biotic and abiotic stresses. Moreover, MIAs play crucial roles in plant growth and response to stimuli (Eilert et al., 1986; Goossens et al., 2003). Kratom originating from its native range exhibits significant variation in MIA profiles. For example, targeted MIA analyses were conducted on seven *M. speciosa* accessions grown across distinct geographical locations, including three red-vein accessions and one white-vein accession from Thailand, a red-vein accession from Malaysia, a red-vein accession from Bali, and a white-vein accession from Borneo (Boffa et al., 2018). Significant variation was observed in the abundance of mitragynine. Notably, the red-vein Malaysian accession contained substantially lower mitragynine levels (4% w/w of total alkaloid extract) compared to the green-vein Malaysian variety (59.7% w/w). In contrast, paynantheine, another common kratom MIA, exhibited the opposite trend, with higher levels in Red Malay (23.6% w/w of total alkaloid extract) relative to Green Malay (9.7% w/w) (Boffa et al., 2018). However, contrasting evidence has been reported from studies quantifying targeted MIAs in commercially marketed kratom products labeled as green, red, and white strains. In these products, no significant differences were observed in either total alkaloid content or the concentrations of individual MIAs across strain designations (Huisman et al., 2023). Another study comparing Malaysian and Thai *M. speciosa* accessions revealed pronounced differences in alkaloid composition (Takayama, 2004; Huisman et al., 2023). Thai-origin accessions predominantly accumulated 9-methoxy-Corynanthe-type indole alkaloids, including mitragynine, speciociliatine, and paynantheine. Although these MIAs were detectable in Malaysian accessions, Malaysian *M. speciosa* produced a broader and more structurally diverse alkaloid profile, including several minor indole alkaloids that are absent or undetectable in Thai accessions. Notably, the relative abundance of mitragynine differed substantially between these accessions: mitragynine constituted 66% w/w of the crude alkaloid content in Thai accessions, whereas it accounted for only ~12% in Malaysian accessions (Takayama, 2004). Even within the same geographical region, there are significant variations in MIA content depending on the season of sample collection. For example, mitragynine, paynantheine and speciogynine contents were compared across 134 samples including the red-vein, and green-vein accessions collected from North, central and Southern provinces in Thailand in June, Oct, and Jan spanning summer and rainy seasons. While the alkaloids in each vein-color type had no significant differences, samples collected during the summer season showed higher mitragynine, paynantheine, and speciogynine than other seasons, with the lowest content in samples collected during rainy season (Sengnon et al., 2023). Although these observations clearly demonstrate environmental influences on MIA composition, they do not investigate the genetic components that may underlie this variation. In addition, of the myriad alkaloids accumulated in kratom, few were targeted for quantitation in these studies, making it difficult to understand the compositional shifts observed in a broader biochemical context. As such, the in-depth study of accessions derived from a defined center of origin is integral to define the extent of intraregional genetic variation and its influence on MIA composition.

DNA barcoding utilizes standardized genomic regions that may allow species-level identification of plant biodiversity. Previous studies validated the *nuclear internal transcribed spacer* (*ITS*) region as a reliable DNA barcode for distinguishing the four *Mitragyna* species native to Thailand (*M. speciosa*, *M. diversifolia*, *M. hirsuta*, and *M. rotundifolia)* enabling inter-specific differentiation within the genus (Sukrong et al., 2007). In addition to *ITS-*based species identification, chloroplast barcode regions, including *ribulose-1,5-bisphosphate carboxylase/oxygenase large subunit (rbcL)*, maturase K (*MatK)*, and *tRNAHistidine (trnH)-Photosystem II protein A (psbA) intergenic spacer (trnH-psbA)*, have also been used alongside the *ITS* barcode to assess sequence variation in commercial kratom powders (Graham et al., 2024). These markers were applied to kratom material sourced from Thailand and Indonesia, where two haplotypes were detected among kratom products (Graham et al., 2024). However, the genotypic diversity observed in products likely reflects the heterogeneous sourcing of plant materials such as harvesting leaves from multiple trees or other species as well as potential postharvest mixing during processing and distribution. Primarily, DNA barcoding research has focused on identifying species-level variations or on evaluating variation across commercial kratom products obtained from broad mixed origins rather than from controlled, tree level sampling within a locality. We have previously conducted DNA barcoding using *ITS* and phylogenetic analysis of a *Mitragyna* germplasm and were able to successfully distinguish not only *Mitragyna* species but also identify inter-specific hybrids involving *M. speciosa* (Laforest et al., 2023). In addition, we associated polymorphisms in *ITS* sequences with MIA profiles indicating *M. speciosa* accessions in our germplasm were from unknown/mixed origins.

In this study, our objective was to investigate the intraspecific genetic variation possible within *M. speciosa* by sampling individuals grown from seeds with differing geographic origins within Thailand and determine whether this genetic variation correlates with targeted MIA profiling. We have characterized eight *M. speciosa* accessions originating from two provinces in Thailand, two (K1&K4) from Pathum Thani, central Thailand, and six (K2&K3, K5-K8) from Trang in southern Thailand. Central Thailand is characterized by a tropical savanna climate and extensive alluvial plains of the Chao Phraya basin, whereas Southern Thailand is a narrow peninsula with a humid tropical monsoon to rainforest climate flanked by eastern and western coastlines separated by mountain ranges (Khedari et al., 2002). We performed DNA barcoding on one individual of each of these eight accessions of *M. speciosa* (K1-K8) using four loci: nuclear *ITS*, and three plastid regions *rbcL, MatK*, and *trnH-psbA* to show intra-specific variation categorizing the accessions into two haplotypes. We have performed targeted alkaloid quantification from K1-K8 *M. speciosa* accessions including seventeen MIAs and oxindole alkaloids and categorized based on C3 stereochemistry from two leaf developmental stages. We further correlated the genetic and developmental chemotype data sets to inform the observed variation in Thai kratom accessions. This framework is helpful for conservation and biodiversity management efforts as well as for authentication and quality control of plant-derived natural products.

## 2 Materials and methods

### 2.1 Plant material and growth conditions

*Mitragyna speciosa* accessions K1–8 were acquired as seeds from Thailand(details provided in **Supplementary Table 1**). The seeds were soaked overnight, poured over a starter tray filled with wet PRO-MIX BX (Premier Tech Horticulture, Quakertown, PA, USA), covered by domes until germination and grown in a greenhouse under a light intensity of ∼500−600 *μ*mol m^−2^ s ^−1^ and an average temperature of 24 °C. After root systems were fully established, seedlings were transplanted into 6inch pots. Following a five-month growth period, the plants were subsequently transferred to 1-gallon containers to support continued growth and development. Fertilization was applied through fertigation using Peters Professional 20-10–20 at a concentration of 100 ppm, and plants were watered daily. Light measurements were taken with a Weston 756 illumination meter, and night interruption of 4.5 *μ*mol m^−2^ s^−1^ for 4 h was implemented that showed improvement in the growth of plants during short days from October to March (Flores et al., 2021; Laforest et al., 2023).

### 2.2 DNA barcoding of *Mitragyna* germplasm

Genomic DNA was obtained from flash-frozen young leaves of one individual per accession K1-K8 of *M*. *speciosa*, and ground into a fine powder using liquid nitrogen. We used a modified hexadecyltrimethylammonium bromide (CTAB) protocol optimized specifically for *M. speciosa* (Laforest et al., 2023). Each sample was combined with 500 μL of extraction buffer (0.35 M sorbitol, 0.1 M Tris-HCl, pH 8.0, 5 mM EDTA, pH 7.5; preheated to 65 °C) that included 0.3% v/v *β*-mercaptoethanol, 1% w/v polyvinylpyrrolidone (PVP) 40,000, and 300 μL of nuclear lysis buffer (0.2 M Tris-HCl, 0.05 M EDTA, pH 8.0, 2 M NaCl, 2% w/v CTAB). The samples were incubated at 65 °C for one hour with gentle inversion every 20 minutes. After cooling to room temperature, 800 μL of chloroform-isoamyl alcohol (24:1) was added and mixed by inversion. Samples were then centrifuged at 13,000 rpm for 15 minutes at room temperature, and the clear aqueous (top) layer was collected. To the final recovered aqueous solution, 0.5 volumes of 6 M NaCl and 0.1 volume of 3 M KCl were added and mixed well. DNA was precipitated using 500 μL of ice-cold isopropanol, incubated at -20 °C for 30 minutes, then centrifuged at 13,000 rpm for 10 minutes. DNA was pelleted, washed with 500 μL of 70% cold ethanol, and centrifuged at 13,000 rpm for 5 minutes. The resulting pellet was dried and resuspended in nuclease-free molecular-grade deionized water.

The nuclear ribosomal *ITS* barcode was amplified from the genomic DNA usingPhusion high-fidelity DNA polymerase (New England BioLabs, Ipswich, MA, USA). The plastid barcodes*MatK, rbcL*, and *trnH-psbA* were amplified using Q5 High-Fidelity 2X Master Mix (New England BioLabs, Ipswich, MA, USA). Primers used in this study are provided in **Supplementary Table 2**. The PCR products were purified using the Wizard SV gel and PCR clean-up system (Promega,Madison, WI, USA), and 60−100 ng of purified PCR product was submitted for Sanger sequencingto GeneWiz according to their submission guidelines. The resulting sequences were aligned and analyzed using Snapgene ‘Align to Reference’ tool (SnapGene 8.2.0). *ITS* of K1-K8 were aligned to *M. speciosa* cultivar 1, GenBank accession: OQ244095, *M. hirsuta*, GenBank accession: OQ244103, and *M. parvifolia*, GenBank accession: OQ244104. The plastid sequences were aligned to the *M. speciosa* plastid genome, accession GenBank: KY085908.1. The distinct *trnh-psba* sequences were further aligned to *M. speciosa* (LC334417.1)*, M. parvifolia* (JX856909.1)*, M. diversifolia* (LC334418.1)*, M. rotundifolia* (LC334419.1)*, and M. hirsuta* (LC334420.1) barcodes available in NCBI GenBank. Sequencing results for all barcodes are provided in **Supplementary Table 3**.

### 2.3 Extraction of targeted MIAs and oxindoles from leaf tissues of *M. speciosa*

Leaves from three biological replicates of each *M. speciosa* accession, K1-8, were flash frozen, and stored at –80 °C. Leaf tissues were ground into a fine powder using liquid nitrogen and stored at −80 °C until needed. Leaf developmental stages were categorized based on size and position: juvenile leaves (<4 cm at the terminal apical meristem) and mature leaves (collected from the main stem, two nodes below the apical meristem) according to (Laforest et al., 2023). Both developmental stages were collected simultaneously to minimize collection related effects. Before alkaloid extraction, ground tissues were lyophilized in a benchtop freeze-dryer (FreeZone, Labconco, Kansas City, MO, USA) for 24 hours at approximately 0.5 mbar and −90 °C to obtain dry weights. A 50 mg aliquot of lyophilized tissue was extracted with 1 mL of 100% ethanol and sonicated for 90 minutes. The solution was centrifuged at 13600 rpm for 1 minute, and the extracts were separated from the insoluble material by pipetting off the supernatant and filtering with a syringe filter (0.2 μm pore; Sigma-Aldrich). For each sample, the process was repeated twice for a total of 3 extractions for complete alkaloid extraction. Filtrates obtained from all three extractions were pooled, and the solvent was subsequently evaporated to concentrate the samples using a nitrogen-line evaporator at 37 °C. The recovered extracts were then lyophilized to complete dryness for 24 hours at approximately 0.5 mbar and −90 °C. The dried fractions were precisely weighed to determine the total crude extract and 2–3 mg of dried extract were used for further analysis.

### 2.4 Identification and quantification of alkaloids by UPLC-MS/MS

The identification and quantification of targeted alkaloids were carried out utilizing a Waters Acquity Class I ultraperformance liquid chromatography (UPLC) system paired with a XevoTQ-S Micro triple quadrupole mass spectrometer (Milford, MA, USA) for the simultaneous quantification of 17 MIAs and oxindole alkaloids. Standards for mitragynine, speciogynine, speciociliatine, mitraciliatine, 7-hydroxy mitragynine, corynantheidine, isocorynantheidine, paynantheine, isopaynantheine, mitraphylline, corynoxine, and corynoxine-B, ajmalicine, speciofoline were previously isolated and purified from dried M. *speciosa* leaves in house at University of Florida (Sharma et al., 2019). 7-hydroxy mitragynine and 9-hydroxycorynantheidine were semi synthesized as described in (Chiang et al., 2024). Strictosidine was gifted by Dr. Yi Tang, and isolated as described in (Misa et al., 2022). Hirsutine was purchased from Sigma-Aldrich (SML3507). All information pertaining to source of standards used, LC-MS/MS transition, retention time, and linearity range are provided in **Supplementary Appendix A1**. These alkaloids were characterized using proton and carbon nuclear magnetic resonance (1H NMR, 13C NMR), high-performance liquid chromatography−photometric diode array (HPLC−PDA), and ultraperformance liquid chromatography–quadrupole time of flight mass spectrometry (UPLC–QTOF) to confirm purity before use in quantitation (Sharma et al., 2019). The original method (Sharma et al., 2019) was slightly modified to reduce the total analysis time per sample. Samples were processed and diluted as previously outlined, and 2 μL of crude alkaloid extract in methanol−water (50:50, %v/v) was injected for chromatographic separation on a Waters Acquity BEH C18 column (1.7 μm, 2.1 × 100 mm). An aqueous ammonium acetate buffer (2.5 mM, pH 3.5) (A) and acetonitrile (B) were used as the mobile phase, with a flow rate of 0.35 mL/min. the gradient program began with 80% A for 1 min, followed by a linear decrease to 77% at 6 min, then a further linear reduction to 10% within 2 min, where it was held for 0.5 min. The column was then re-equilibrated to the initial condition (80% A) for 0.7 min. Data acquisition and analysis were performed using MassLynx XS version 4.1. Alkaloid quantities are reported as mass percent analyte per dried tissue extracted.

### 2.5 Statistical analyses

Statistical analyses were performed using SAS JMP (SAS Institute, Inc., Cary, NC, United States). First, Huber and quartile methods were used to evaluate data for outliers. Alkaloid quantities below the lower level of quantification and outliers were treated as missing for statistical analysis. For each alkaloid per accession, normality was assessed using the Shapiro-Wilk test for normality. Values were adjusted as follows to normalize data: ln(isocorynantheidine) in K2, ln(corynantheidine) and inverse mitragynine in K3, ln(corynantheidine) in K4, inverse mitragynine in K6, and ln(speciociliatine), ln(mitraciliatine, and ln(isopaynantheine) in K8. Homogeneity of variance across accessions was assessed with Levene’s test. Comparison between overall 3*S* and 3*R* accumulation in mature and juvenile leaves, and comparisons of mature and juvenile alkaloid content for each accession were assessed using two-tailed t-tests. To determine if accession and/or tissue type had significant effects on leaf alkaloid content, fixed effects for each alkaloid was estimated using a mixed model (Type III REML) across accessions K1-K8. Comparison of alkaloid content across accessions within developmental groups was done using ANOVA or Welch’s test as appropriate based on homogeneity of variance. For alkaloids for which there were significant differences of means across accessions, Tukey’s Honestly Significant Difference *Post-hoc* test was used to identify significantly different groups. Statistical tests were considered significant if *P* ≤ 0.05.

## 3 Results

### 3.1 Morphological characterization of Thai kratom accessions (K1-K8) focusing on leaf shape, margin variation, and vein coloration

**Figure 1:**
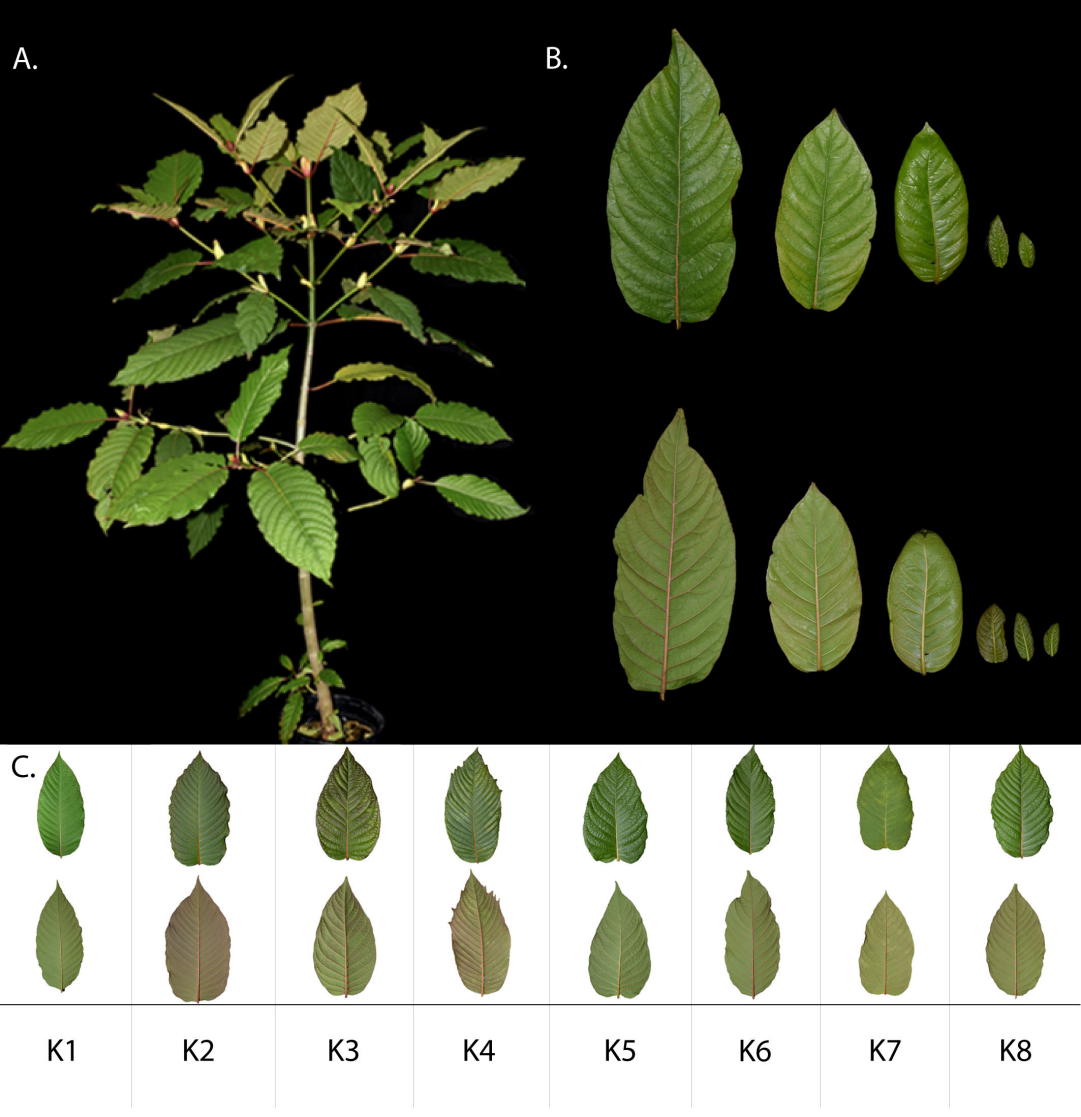
Morphological differences in mature leaves of *M. speciosa* accessions K1-8. **(A)** *M. speciosa* (K8) tree, mature leaves originating from the main stem **(B)** Adaxial and abaxial sides of mature and juvenile leaves of *M. speciosa* accession K8. Leaf vein coloration is variable between leaves of different developmental stages ranging from bright red in mature leaves to pale green veins in juvenile leaves within the same tree collected at one timepoint. **(C)** Variation in leaf color, vein number, leaf blade shape, and tip morphology in K1-8. Adaxial surface shown above the corresponding abaxial surface.

Kratom leaf vein coloration is widely recognized and frequently used in the commercial classification and marketing of kratom leaf products. However, our observations indicate that one individual tree may have variable vein color depending on the leaf developmental stage. For example, Thai accession K8 (**Figure 1A**) produces leaves exhibiting the full range of vein coloration spanning from green to red within a single tree as shown in (**Figure 1B**). The Thai K1-K8 accessions displayed considerable leaf morphological variation, with leaf shapes ranging from elliptic (K1, K6) to ovate (K2-K5, K7-K8), and leaf margins ranging from entire/smooth (K1, K3, K7) to undulated/ruffled (K2, K5-6, K8). Most leaves exhibited acuminate apices except K4, which had a serrated apex. Mature leaves further differed in venation, containing 10–20 primary veins with vein coloration of the leaves ranging from light green (K5) to red (K2) (**Figure 1C**). Leaf morphology was consistent across the developmental stages and replicates within a given accession, with exception of vein coloration. We further sought to integrate the observed morphological variation, including differences in leaf vein coloration, overall leaf pigmentation, and leaf margin morphology with DNA barcoding and targeted MIA analyses to assess whether the phenotypic diversity among samples has an underlying genetic basis.

### 3.2 Molecular characterization using DNA barcoding identifies intra-specific diversity and distinct haplotypes in kratom accessions

The *ITS* universal barcoding region was successfully amplified for all Thaiaccessions K1-K8. Sanger sequencing produced amplicons of 502 bp for all accessions. No sequencevariations were detected among K1-K8 relative to the vouchered *M. speciosa* (accession OQ244095) (**Supplementary Table 3**; **Supplementary Figure 2**). The *ITS* barcode is known to provide good species-level resolution; however, it may not show sequence differences among individuals within a species as it is conserved (Chase and Fay, 2009; Fazekas et al., 2009; Roy et al., 2010; Cheng et al., 2016). Therefore, additional markers including chloroplast DNA barcodes *rbcL* and *MatK*, from coding regions, and *trnH-psbA* from a non-coding intergenic spacer were also amplified from kratom accessions K1-K8. While *rbcL* also had no sequence variations among K1-K8 accessions, *MatK* and *trnH-psbA* contained sequence variations distinguishing them. For the *matK* barcode, three single nucleotide polymorphisms (SNPs) were identified in accessions K5 and K6, including G/C, G/A and a G/T SNPs at 2550bp, 2786 bp and 2877 bp and these variants were consistent between the two accessions (**Table 1**). Similarly, for the *trnH-psbA* barcode, six sequence variations were identified in the same K5 and K6 individuals including in-dels and SNPs. These included, a ‘T’ deletion at 93 bp, a A/T SNP at 94bp, a 3bp deletion at 95bp, a ‘TG’ insertion at 111bp, an ‘A’ deletion at 192 bp, and a 9bp deletion at 338 bp. The sequence variations observed in K5 and K6 were identical, while no differences were detected in the remaining accessions, thereby resolving the samples into two distinct haplotypes (**Table 1**). Alignments of our alternative haplotype (K6) and available *trnh-psba*barcoding loci for five *Mitragyna* species revealed that all these polymorphic sites were identical between *M. diversifolia, M. rotundifolia*, *M. hirsuta*, and K5 and K6 (**Supplementary Figure 1**). Alignments of K1–8 against available *ITS* barcoding of *M. hirsuta* and *M. parvifolia*, however, showed no shared SNPs or indels with any of the accessions (**Supplementary Figure 2**).

**Table 1:** DNA barcoding using chloroplast barcodes identified polymorphisms across K1-K8 kratom accessions.

K8	G	G	G	T	A	ATable	–	A	ACAAATAAG
K7	G	G	G	T	A	ATable	–	A	ACAAATAAG
K6	C	A	T	–	T	–	TG	–	–
K5	C	A	T	–	T	–	TG	–	–
K4	G	G	G	T	A	ATable	–	A	ACAAATAAG
K3	G	G	G	T	A	ATable	–	A	ACAAATAAG
K2	G	G	G	T	A	ATable	–	A	ACAAATAAG
K1	G	G	G	T	A	ATable	–	A	ACAAATAAG
Polymorphism	G/C SNP	G/A SNP	G/T SNP	T’ Deletion	A/T SNP	3bp Deletion	TG Insertion	A Deletion	8bp Deletion
Position	2550	2786	2877	93	94	95-97	111-112	191	338-346
DNA barcode	*matK*	*matK*	*matK*	*trnH-psbA*	*trnH-psbA*	*trnH-psbA*	*trnH-psbA*	*trnH-psbA*	*trnH-psbA*

Colored text is used to emphasize the SNPs and indels that differentiate kratom accessions K1-K8 into two haplotypes.

### 3.3 Developmental variation in monoterpene indole alkaloid accumulation across Thai K1-K8 *M. speciosa* germplasm

**Figure 2:**
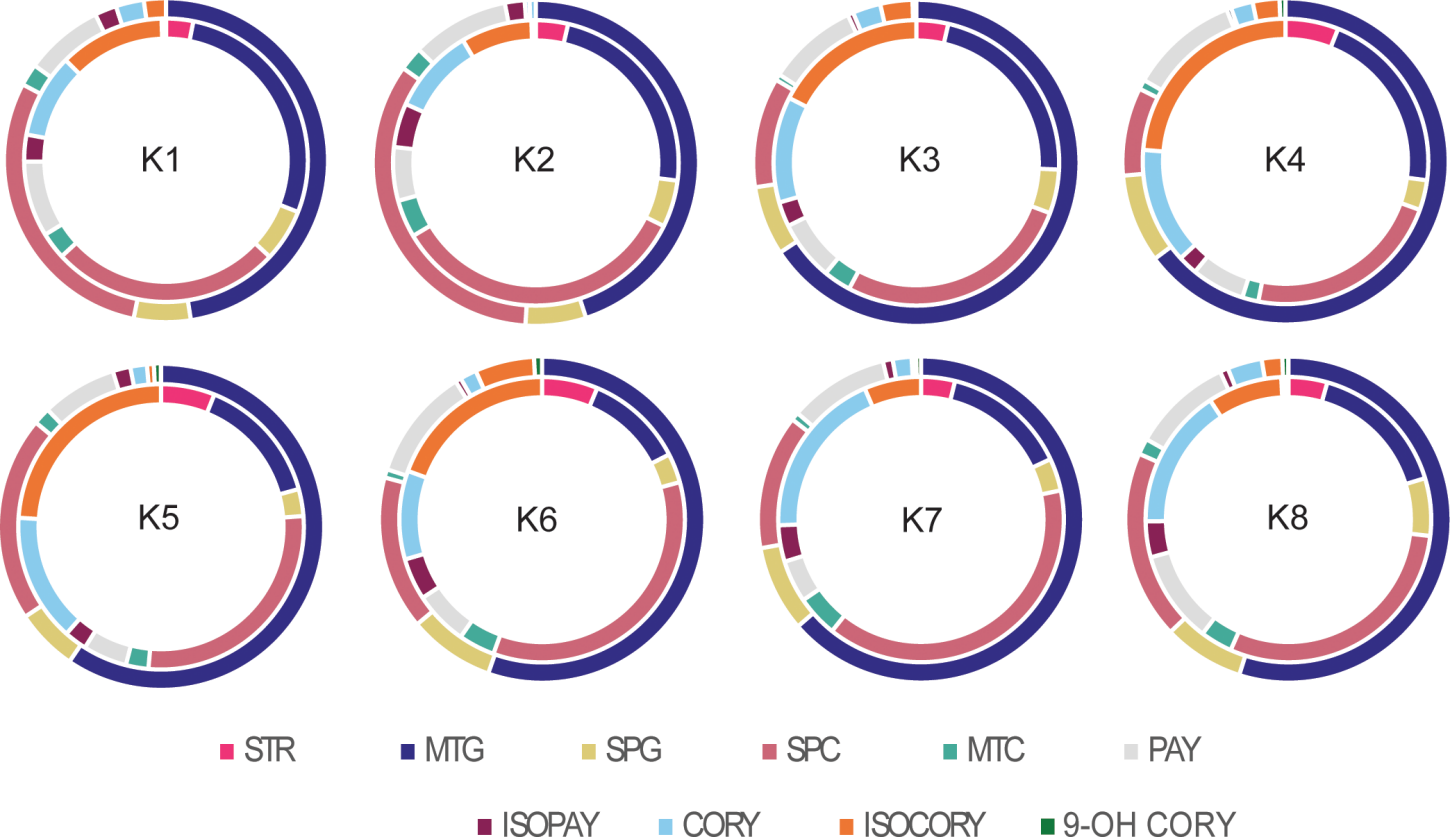
Quantification of targeted monoterpene indole alkaloids (MIAs) in juvenile and mature leaves of*M. speciosa* accessions K1-8. Donut diagrams represent the absolute quantities ofMIAs based on %weight/leaf dry weight. The outer ring represents the mature leaf profile, and the inner ring represents the juvenile leaf profiles. Strictosidine (STR), mitragynine (MTG), speciogynine (SPG), speciociliatine (SPC), mitraciliatine (MTC), paynantheine (PAY), isopaynantheine (ISOPAY), corynantheidine (CORY), and isocorynantheidine (ISOCORY), and 9-hydroxy corynantheidine (9-OH COR). The absolute quantification values for all metabolites are included in **Supplementary Table 4**. Significant differences between 3*S* and 3*R* MIAs in juvenile and mature leaves are denoted as ***p < 0.001 and ns, not significant.

**Figure 3:**
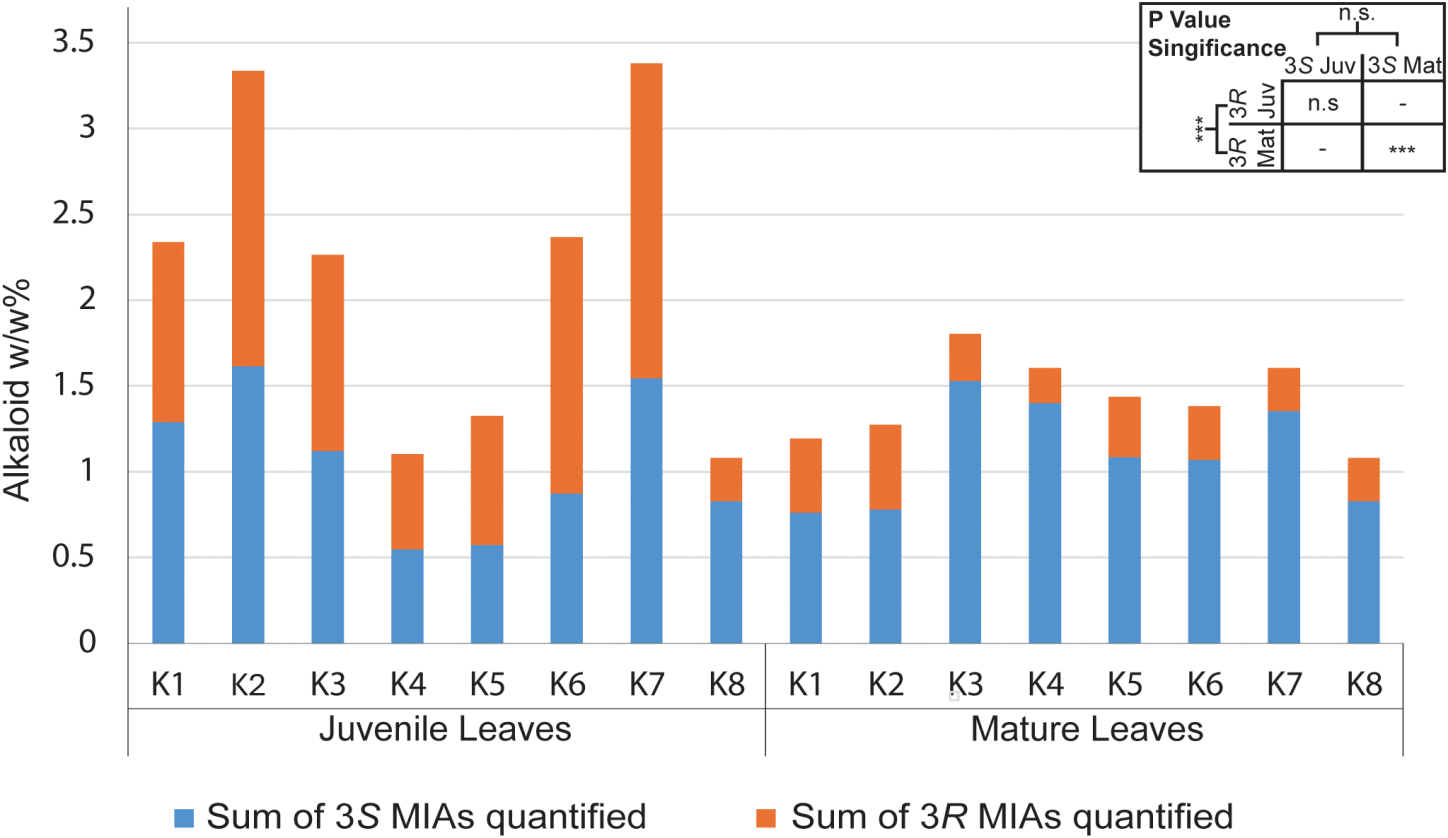
Bar chart illustrating the relative ratio of 3*S* versus 3*R*monoterpene indole alkaloid stereoisomers in mature and juvenile leaves from *M.speciosa* accessions K1-K8 as a % weight/leaf dry weight. 3*S* alkaloids in blue constitute the sum of strictosidine, mitragynine, speciogynine, paynantheine, corynanthedine, and 9-hydroxy corynanthedine. 3*R* alkaloids in orange are the sum of speciociliatine, isocorynantheidine, mitraciliatine, hirsutine, and isopaynantheine. The absolute quantification values for all metabolites correspond to **Supplementary Table 4**.

We targeted quantification of seventeen MIAs and oxindole alkaloids from two leaf developmental stages, juvenile (<4cm) and mature leaves following previously established guidelines for developmental stage determination (Laforest et al., 2023) across K1-K1-8 accessions of Thai *M. speciosa.* Ten of the targeted alkaloids were detected and quantified (**Figure 2**), while the remaining compounds were below the detection limit (**Supplementary Table 4**). We observed both qualitative and quantitative shifts in the developmental profiles ofjuvenile and mature leaf MIAs (**Supplementary Table 3**). The central MIA precursor, strictosidine was detected exclusively in juvenile leaves,remaining below the detection limit in mature leaves regardless of the accession (**Supplementary Table 4**). Similarly, corynantheidine, proposed as a precursor of mitragynine in the MIA pathway,accumulated at higher levels in juvenile leaves than in mature leaves (**Supplementary Table 4**). The alkaloid profile of young leaves was dominated by speciociliatine (up to 1.32% w/wleaf dry mass), the highest accumulating MIA followed by corynantheidine (up to 0.65% w/w leaf drymass), mitragynine (up to 0.50% w/w leaf dry mass), and iso-corynantheidine (up to 0.46% w/w leaf dry mass) (**Supplementary Tables 4**, **5**). Although mitragynine levels in the juvenile leaves were comparable across all accessions, most showed an increase in mitragynine content in mature leaves, except for K1 and K2 where juvenile mitragynine content was on par with mature leaves (**Figure 2**; **Supplementary Tables 4**, **5**). Furthermore, the accumulation of their upstream precursors, including strictosidine, corynantheidine, and isocorynantheidine decreased in mature tissues (**Figure 2**; **Supplementary Tables 4**, **5**). Alkaloids corynoxine, corynoxine-B, mitraphylline, ajmalicine, 9-hydroxycorynantheidine,hirsutine, and speciofoline were below the lower limit of quantification (**Supplementary Table 4**). The total alkaloid content calculated as the sum of all targeted alkaloids quantified was higher in juvenile leaves ranging from 2.26% - 3.38% w/w, compared with mature leaves 1.08%-1.8% w/w across all accessions, with the exception of K4 and K5 (**Figure 3**).

Given that the significant stereoselectivity at C3 and C20 generates distinct 3*S* and 3*R* diastereomers, and our earlier analyses showed developmental shifts in their relative abundance, we examined their distribution in juvenile and mature leaf tissues. While the overall alkaloid content generally decreased in mature leaves, 3*S*/3*R* stereochemical distribution had an inverse relationship upon maturation, where juvenile leaf tissue was dominated by 3*R* MIA isomers, whereas mature leaves accumulated higher levels of 3*S* alkaloids (**Figure 3**). We examined the variations in MIA profile among accessions K1-K8 from varying geographical regions within Thailand. Although mature leaves display considerable morphological variation, including differences in vein coloration (**Figure 1C**), no significant differences were detected in alkaloid profiles across accessions,regardless of vein color and morphology (**Supplementary Tables 4**, **6**, **7**). On the other hand, significant variations across *M. speciosa* accessionswere observed in MIA accumulation in juvenile leaves for most detected compounds, except theprecursors strictosidine, corynantheidine, and iso-corynantheidine (**Supplementary Tables 6**, **7**). Overall, development rather than accession had a significant effect on MIA accumulation,apart from speciogynine and paynantheine, for which the interaction of development and accession hada significant effect (**Supplementary Table 8**).

## 4 Discussion

Kratom leaves are the primary plant part utilized in commercial kratom products. These products are often powders, marketed in a range of colors from green to red, that are reported to have different effects on the consumer. Given this variation, we investigated whether vein color variation or other leaf morphological variations present in Thai *M. speciosa* accessions K1-K8 have an underlying genetic basis and whether differences in alkaloid profiles are associated with this leaf morphology variation. The *ITS* barcode exhibited no sequence variations among K1-K8 Thai individuals when aligned against *M. speciosa* (GenBank OQ244095), and several polymorphic sites when compared against *M. hirsuta* and *M. parvifolia* (**Supplementary Figure 2**). This result is consistent with the findings of (Sukrong et al., 2007), who reported that all ten *M. speciosa* samples collected from different geographic locations, despite exhibiting morphological variation, shared an identical *ITS* barcode region. A previous study reported multiple polymorphisms in the *ITS* region among geographically diverse *M. speciosa* accessions (Graham et al., 2024). However, their study relied on commercial kratom powders, which have unknown or mixed genetic backgrounds and may contain contaminants, potentially contributing to the observed polymorphisms.

We used additional DNA barcodes when *ITS* showed no polymorphisms between *M. speciosa* accessions (**Supplementary Figure 2**), as relying on a single genetic marker is often insufficient to capture true biological and evolutionary relationships among samples. For plants, DNA barcodes often exhibit variance in discriminatory power based on lineage, and barcode combinations, specifically of the *ITS*, *rbcL, matK*, and t*rnH-psbA* greatly improve species differentiation (CBOL Plant Working Group et al., 2009; Pang et al., 2012; Techen et al., 2014). In some instances, plastid genes have been shown to resolve taxonomic relationships within groups with poor sequence divergence (Amar, 2020). In addition, plastid barcodes can reveal lineage structures that *ITS* cannot, since plastid genomes retain lineage-specific signatures due to uniparental inheritance (Hernández-Verdeja, 2026).

Therefore, we used plastid DNA barcodes *rbcL, matK*, andt*rnH-psbA*, the preferred combinatorial barcoding regions for land plants (CBOL Plant Working Group et al., 2009) to detect and identify polymorphisms among the K1-K8 accessions. While *rbcL* did not have any sequence variations, *trnH-psbA* and *matK* successfully distinguished intra-specific variation in the Thai *M. speciosa* germplasm, suggesting the combination may be apt for evaluating intra-specific relationships in this genus. Interestingly, the polymorphisms detected in both plastid barcodes were identical in K5 and K6, while all remaining accessions shared the reference sequence, clearly resolving the collection into two distinct haplotypes. Because both markers yielded congruent results, the presence of two haplotypes in this set of accessions can be inferred with high confidence. However, there was no distinct correlation between haplotypes and geographic origin. Although K5 and K6 accessions both were collected from southern Thailand, four other accessions from that region did not possess this haplotype. Instead, their DNA barcodes were identical to those of the accessions collected from central Thailand. Our study suggests that while *ITS* is an accepted nuclear DNA barcode to identify inter-specific variations, it is insufficient for identifying intra-specific variations in *M. speciosa.* We showed that using multiple plastid DNA barcodes may be best at identifying intra-specific variations and providing better resolution. Though our study found no differences in *ITS*, previous work found two haplotypes using this region, as well as variations in *matK* and *trnH-psbA*. However, their work was based on commercial powders which may comprise mixtures of tissues (Graham et al., 2024). In a barcoding analysis carried out on leaves of vouchered trees, shared variations in the *trnH-psbA* barcode were reported between *M. diversifolia, M. rotundifolia*, and *M. hirsuta*, but those polymorphisms were not found in *M. speciosa* (Jaipaew et al., 2018). Supporting this, genome resequencing of 85 *M. speciosa* accessions previously identified two distinct sub-populations of *M. speciosa* in central and southern Thailand (Pootakham et al., 2022). Here, we show that some kratom accessions share the polymorphisms previously undetected in the *Mitragyna speciosa trnH-psbA* barcode (**Supplementary Figure 1**). One possibility is that this barcoding region may capture the subpopulation diversity previously described in Thai *M. speciosa*, not hybridization, supported by analysis of nuclear barcoding regions. Notably, our study is limited to few accessions, and expanded characterization across a larger population would be beneficial in understanding the genetic structures captured by the *trnH-psba* marker. Although two distinct haplotypes were identified in our data, leaf morphology alone did not allow us to differentiate K5 and K6 from other accessions. For example, while K5 and K6 exhibited ruffled leaf margins, K8 also shared this trait. Similarly, vein coloration was green in K5 but red in K6, and both colors occurred in other accessions in the collection. Thus, neither margin type nor vein color reliably corresponds to haplotype identity. Therefore, we conducted targeted MIA profiling to determine whether the chemical composition correlates with haplotype identity and to assess whether the two accessions belonging to the same haplotype exhibit matching alkaloid profiles.

Targeted MIA profiling clearly indicated prominent developmental variation in *M.speciosa* leaf alkaloid content and composition, with differences between juvenile andmature leaves being relatively conserved in patterning (**Supplementary Tables 5**, **8**). All accessions showed consistent MIA profiles in their mature leaves with no significantvariations (**Supplementary Table 5**). While we observed variation in vein coloration within and across *M. speciosa* accessions (**Figures 1B, C**), no correlation was found between vein color and alkaloid profile. In fact, leaves of the same tree varied in vein coloration (**Figure 1B**) depending on the stage of development. This result is congruent with previous studies that examined vein coloration and alkaloid accumulation, showing the two are uncorrelated regardless of season or geographical differences (Sengnon et al., 2023). In addition, surveys of kratom users revealed that vein color-based effects reported reflected marketing narrative rather than alkaloid composition (Huisman et al., 2023). Here, we present evidence that these conclusions, evaluated in a Thai *M. speciosa* germplasm, remain true in fresh, flash frozen tissue and for a large set of MIAs.

Notably, no significant differences in alkaloid accumulation were detected in mature tissues across accessions, or between haplotypes. While this could imply that these accessions are ultimately similar in genotypic control of alkaloid content, it is important to note that secondary metabolites and environment are inextricably linked. A recent study evaluating effects of different controlled environment factors on *M. speciosa* grown from seed found that light intensity and water conditions significantly influenced alkaloid content in leaves (Leksungnoen et al., 2026). In addition, in a survey of *M. speciosa* trees growing across Thailand, alkaloid content was found to be significantly associated with an environmental gradient, with light intensity and relative humidity having strong correlations with alkaloid content (Leksungnoen et al., 2022). As alkaloid accumulation is a plastic trait, growing all accessions K1-K8 under uniform conditions may have masked variations in response to environmental cues that would otherwise be detected across kratom trees in their native habitats.

While the downstream alkaloids, particularly mitragynine, showed significant increases in mature leaves, their upstream precursors, including strictosidine, corynantheidine, and isocorynantheidine, declined with leaf maturation. Notably, strictosidine, the central precursor of MIA biosynthesis, was detected exclusively in juvenile leaves. The relative distribution of 3*S* and 3*R* stereoisomers remained consistent across accessions and haplotypes and the biochemical shift from 3*R* to 3*S* along a developmental axis is congruent with previous findings (Laforest et al., 2023). Across all accessions, juvenile leaves were dominated by 3*R* MIAs with the majority of the differences being attributable to speciociliatine and isocorynantheidine content, whereas mature leaves accumulated higher levels of 3*S* MIAs (**Figure 3**, **Supplementary Table 4**). This coordinated decline in precursors, rise in downstream alkaloids, and switch in stereochemical profiles collectively suggests precursor utilization during leaf maturation and that young leaves constitute a more active MIA biosynthesis site. Supporting this, the recently discovered enzymes involved in generating 3*R*MIAs in *M. speciosa* were found to be highly expressed in young leaves, along with the majority of the elucidated MIA pathway genes (McDonald et al., 2025). The variation of individual downstream MIA levels observed among juvenile leaves, for example mitragynine in K1 and K2 may in part reflect inconsistencies in sample collection. Although we defined juvenile leaves as those <4 cm in length at the terminal apical meristem, this criterion may not correspond to the same developmental stage across all accessions, given potential differences in their leaf expansion rates and maturity. This variation likely reflects natural differences in growth rates and leaf size among accessions, underscoring the importance of developmental stage standardization in comparative MIA studies.

## 5 Conclusions

This study provides a comprehensive characterization of developmental and genotypic influences on MIAs accumulated across eight Thai kratom accessions, offering new insights into the metabolic regulation that underpins alkaloid diversity in *Mitragyna speciosa*. By profiling both early precursors and downstream alkaloids across leaf stages, we demonstrated that MIA metabolism is strongly developmentally programmed, with clear and conserved biochemical patterns across all eight accessions. Juvenile leaves consistently accumulated the highest levels of upstream precursors - including strictosidine, which was uniquely detected at this stage along with elevated pools of intermediate MIAs such as corynantheidine and isocorynantheidine. In contrast, mature leaves displayed higher concentrations of the downstream bioactive alkaloids. The inverse accumulation patterns of precursors and 3*S* MIA end products, coupled with the absence of strictosidine in mature tissues, strongly suggest that young leaves are active sites of MIA biosynthesis, with a highly active 3*R*-directed MIA pathway in juvenile leaves, and reduced 3*R* MIA accumulation in mature leaves. Stereochemical patterns were similarly conserved across accessions, revealing an additional layer of developmental regulation. Juvenile leaves were enriched in 3*R* stereoisomers, whereas mature leaves predominantly accumulated 3*S* forms. This consistent developmental stereochemical switch indicates coordinated control of stereoselective oxidation/reduction steps and may reflect selective pressures favoring distinct alkaloid chemotypes at different physiological stages of leaf development. Despite visible morphological diversity among mature leaves, including distinct vein coloration and margin phenotypes, alkaloid profiles at maturity remained largely consistent across accessions. This suggests that visual leaf traits, although useful for accession identification, are not reliable predictors of MIA chemical composition. Together, these findings highlight a tightly coordinated and developmentally regulated organization of the MIA biosynthetic pathway that must be explored further. They further emphasize that leaf developmental stage is a dominant determinant of alkaloid composition, overshadowing plastid haplotypes and accession differences under the conditions examined. The conserved patterns presented in this study lay a biochemical foundation for targeted breeding, metabolic engineering, and controlled cultivation strategies aimed at optimizing specific MIA profiles, particularly those of pharmacological interest such as mitragynine. Future work integrating transcriptomic, enzymatic, and metabolic flux analyses will be critical to resolving the regulatory nodes that drive these developmental shifts, enabling deeper understanding and manipulation of MIA pathways in *Mitragyna speciosa* and related species.

## Data Availability

All barcodes generated are available in NCBI Genbank. The ITS barcodes are under accession numbers PZ261277-PZ261284, and plastid barcodes trnH-psbA, matK, and rbcL can be found as Genbank accessions: PZ268178-PZ268201.

## Supplementary material

Supplementary Figure 1 MAFFT alignment of *trnh-psba* barcoding sequence for five *Mitragyna* species showing presence of a variable haploblock in Thai *M. speciosa* accession K6.

Supplementary Figure 2 Alignment of *ITS* barcoding sequence of *M. speciosa*(GenBank accession OQ244095), K1-8, *M. hirsuta*, (OQ244103), and *M. parvifolia* (OQ244104) showing differences in SNPs between *M. speciosa* and other *Mitragyna* species.
