# Supplementary figures and images for: Comparative analysis of monoterpene indole alkaloid composition and genotypic variation in Thai Mitragyna speciosa
Source: Front Plant Sci. 2026 May 4;17:1821609. doi: 10.3389/fpls.2026.1821609 (PMC13180935; doi:10.3389/fpls.2026.1821609)

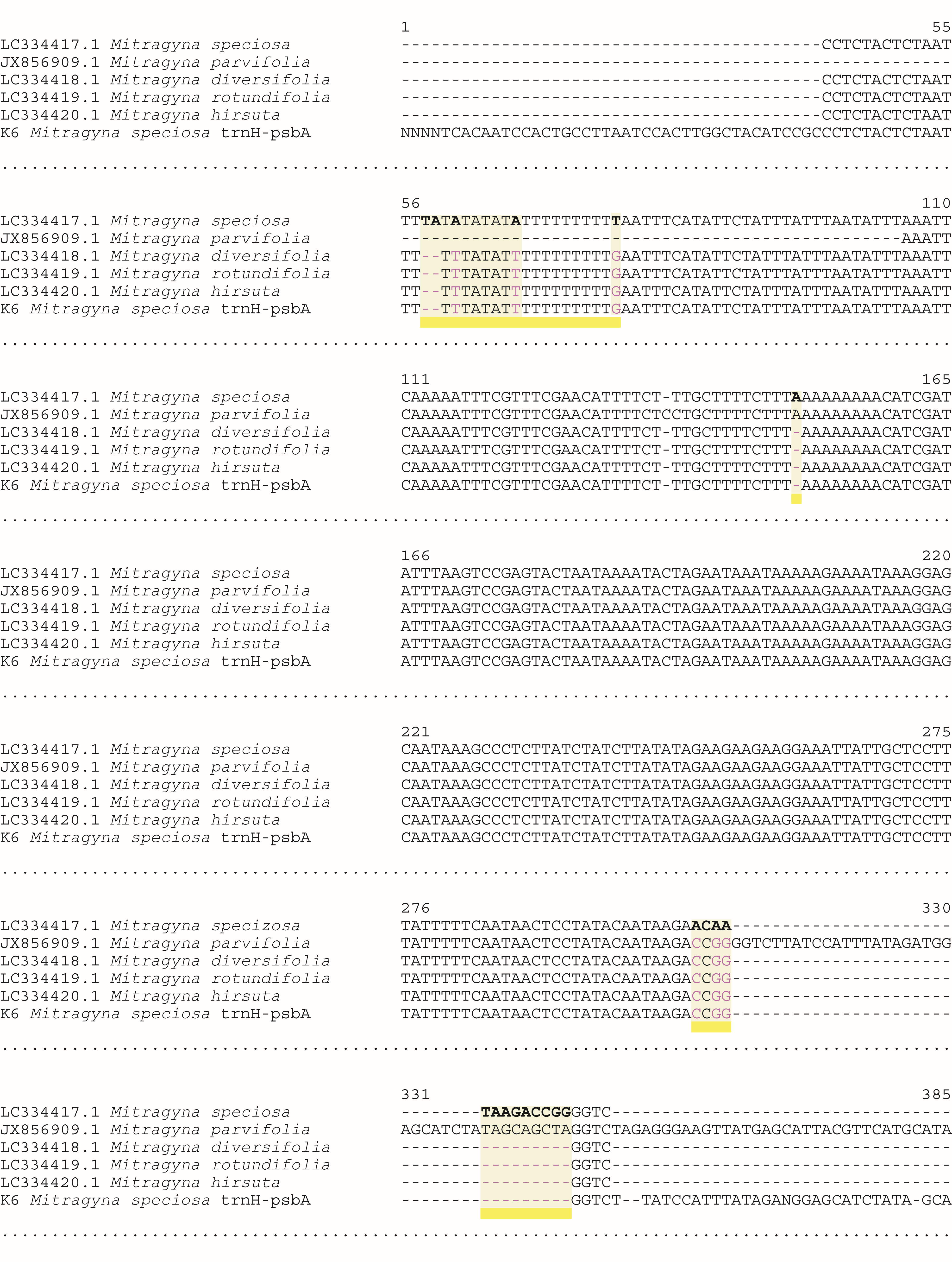

Supplement: Supplementary Figure 1 — MAFFT alignment of trnh-psba barcoding sequence for five Mitragyna species showing presence of a variable haploblock in Thai M. speciosa accession K6. [file SupplementaryFile1.zip › Supplementary Fig 1.TIF]

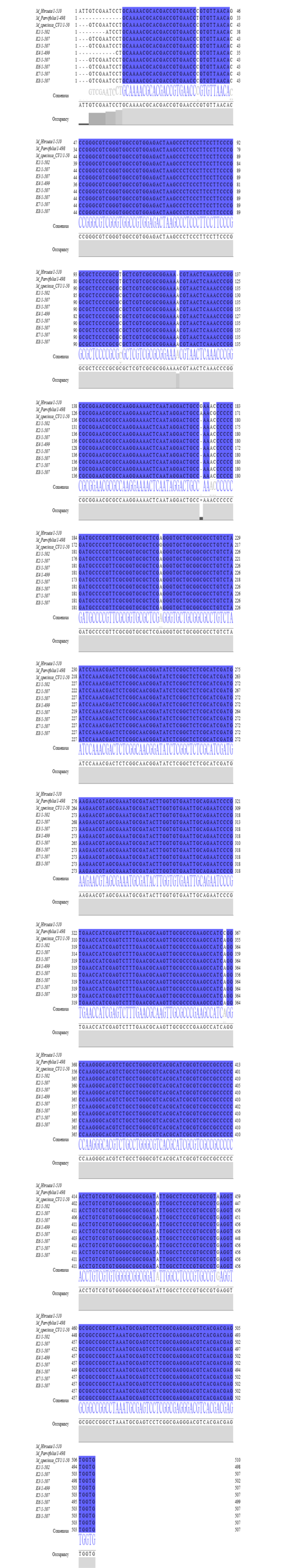

Supplement: Supplementary Figure 1 — MAFFT alignment of trnh-psba barcoding sequence for five Mitragyna species showing presence of a variable haploblock in Thai M. speciosa accession K6. [file SupplementaryFile1.zip › Supplementary Fig 2.tif]
